# Fastphylo: Fast tools for phylogenetics

**DOI:** 10.1186/1471-2105-14-334

**Published:** 2013-11-20

**Authors:** Mehmood Alam Khan, Isaac Elias, Erik Sjölund, Kristina Nylander, Roman Valls Guimera, Richard Schobesberger, Peter Schmitzberger, Jens Lagergren, Lars Arvestad

**Affiliations:** 1 KTH Royal Institute of Technology, Science for Life Laboratory, School of Computer Science and Communication, Department of Computational Biology, Solna, Sweden; 2 Science for Life Laboratory, Department of Biochemistry and Biophysics, Stockholm University, Solna, Sweden; 3 KTH Royal Institute of Technology, School of Computer Science and Communication, Stockholm, Sweden; 4 KTH Royal Institute of Technology, Stockholm Bioinformatics Center, School of Computer Science and Communication, Department of Computational Biology, Stockholm, Sweden; 5 Department of Numerical Analysis and Computer Science, Swedish e-Science Research Centre (SeRC), Stockholm University, Stockholm, Sweden; 6 University of Applied Sciences Upper Austria, School of Informatics, Communications and Media, Department of Medical and Bioinformatics, Hagenberg, Austria; 7 UET University of Engineering and Technology Peshawar, Department of Computer Science and Information Technology, Peshawar, Pakistan

## Abstract

**Background:**

Distance methods are ubiquitous tools in phylogenetics. Their primary purpose may be to reconstruct evolutionary history, but they are also used as components in bioinformatic pipelines. However, poor computational efficiency has been a constraint on the applicability of distance methods on very large problem instances.

**Results:**

We present fastphylo, a software package containing implementations of efficient algorithms for two common problems in phylogenetics: estimating DNA/protein sequence distances and reconstructing a phylogeny from a distance matrix. We compare fastphylo with other neighbor joining based methods and report the results in terms of speed and memory efficiency.

**Conclusions:**

Fastphylo is a fast, memory efficient, and easy to use software suite. Due to its modular architecture, fastphylo is a flexible tool for many phylogenetic studies.

## Background

Distance methods are important for phylogenetic inference, and this is confirmed by the many available algorithms and software implementations [1-12]. The main ambition with several implementation efforts has been to improve the computational efficiency, which is essential for any method’s applicability. In particular, the cubic time complexity of Neighbor Joining (NJ) [13] has been an obvious obstacle that several groups have challenged. One of these efforts is Fast Neighbour Joining (FNJ), a quadratic-time algorithm for tree reconstruction presented by Elias and Lagergren [14]. They showed in [14] that FNJ performs similar to the canonical NJ method. FNJ modifies the NJ selection function for joining any pair of sequences together and introduced the concept of *visibility set* to avoid redundant computation, thus, giving a significant improvement in speed and similar accuracy as NJ for computing the phylogenetic tree. This paper presents fnj, a fast and practical implementation of the FNJ algorithm.

A sometimes overlooked issue in distance-based method development is that the distance matrix, the input to tree reconstruction algorithms, is the real computational bottleneck. With *n* sequences of length *l*, you cannot do better than time *O*(*ln*^2^) for estimating a distance matrix. Since *l* is rarely smaller than *n*, the distance computations have cubic time complexity, and there is therefore little gain with efficient tree reconstruction.

We address this efficiency problem by making speedup techniques by [15] available in a space-efficient implementation through the fastdist program. With novel substitution-counting algorithms and register-based bit-fiddling in 128-bit registers, common distance estimators for DNA sequence can reach a speedup of two orders of magnitude compared to e.g. PHYLIP. In addition, the implementation makes optimal use of ambiguity symbols instead of dismissing them, as described in [15]. Similarly, for fast computation of the distance matrices of protein sequences, we introduce fastprot and fastprot_mpi.

We present fastphylo as a package containing phylogenetic tools of efficiency.

## Implementation

Fastphylo consists of four modules: fastdist, fastprot, fastprot_mpi, and fnj. All these modules are implemented in C++ (compiled with GCC v4.7) and have been verified to compile on popular platforms: Scientific Linux, Ubuntu and Mac OS X. The programs follow classic Unix principles to achieve modularity and composability. This means that the user can decide whether to read from the file(s) or use I/O redirection. In particular, you can construct a Unix pipeline such as

to compute a phylogenetic tree and save it to a file.

By reading and writing the commonly used sequence formats, FASTA and PHYLIP, compatibility is maintained with existing phylogenetic tools such as PHYLIP [4] and RaxML [10]. However, we have also implemented support for XML-based I/O to encourage validatable data handling. Using XML simplifies format conversion, safe-guards against formatting mistakes, and enables validation of input and output. To support validation, the RelaxNG XML [16] schemas for sequence data, distance matrices, and phylogenies are builtin to all the fastphylo modules and can be easily retrieved from the programs. Unlike the PHYLIP format, XML also enables users to work with long accessions.

One of the main issues with phylogeny reconstruction is the storage of distance matrices. It requires a large amount of disk space to store a distance matrix for very large gene families. We, therefore, introduce a binary format that overcomes this problem (see Section 'Features of **fastdist**’ for further details).

### Features of fastdist

The fastdist program estimates distance matrices from DNA alignments. It implements fast computation of four distance estimators: Hamming (also known as *p*-distance), JC [17], K2P [18], and TN93 [19]. K2P is the default distance estimator for fastdist.

**Figure 1:**
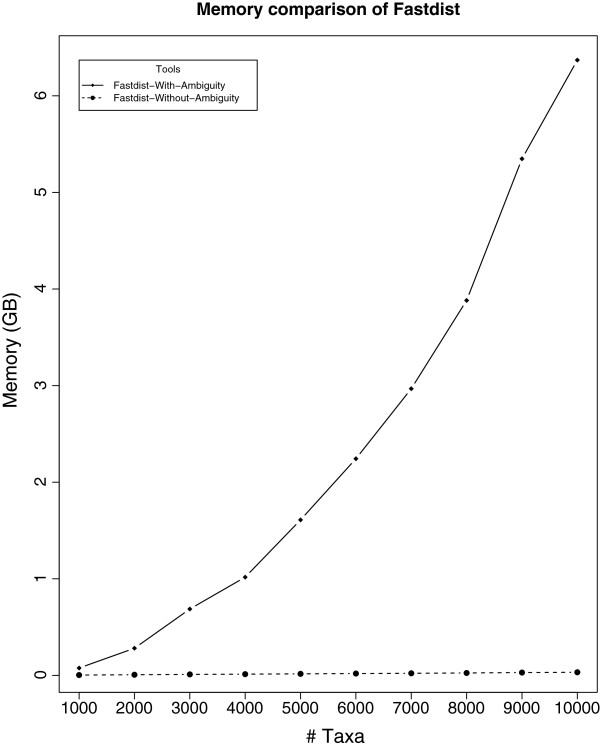
**Memory consumption of ****fastdist**** program.** This figure shows fastdist computation on 10 gene families with family size ranging from 1,000 to 10,000. Here, Fastdist-without-Ambiguity refers to the results computed using binary format functionality (discussed in section 'Features of **fastdist**’), while Fastdist-with-Ambiguity refers to the fastdist computation using ambiguity information. The results in the figure suggest that the Fastdist-with-Ambiguity computation requires much more memory than Fastdist-without-Ambiguity as the gene family size increases.

The two distinguishing features of fastdist, however, are speed and the support for ambiguity symbols (see further [15]). fastdist computes the whole distance matrix using ambiguity symbols in a default mode, which requires quadratic memory space as the gene family size increases (see Figure 1). To overcome this problem, we introduce a binary format that performs row-wise operations in computing the upper triangular distance matrix. Furthermore, instead of keeping the whole distance matrix in plain text, we store the upper triangular matrix in a binary format that reduces the amount of disk space substantially. For instance, the distance matrix computed by fastdist using the binary format for 100,000 sequences, with each sequence of length 2000 bp, took ∼19 GB of disk space while the distance matrix for the same set of sequences computed by RapidNJ [12] using PHYLIP format consumed ∼76 GB of disk space. Using the memory-efficient option, fastdist allows the users to do row-wise operations while computing the distance matrix, i.e., keeping only a single row of the distance matrix in memory. When the binary format option is used, memory-efficient functionality is implicitly invoked. Both memory-efficient and binary format, however, do not support ambiguity symbols information for computing distance matrix.

### Features of fastprot and fastprot_mpi

**Figure 2:**
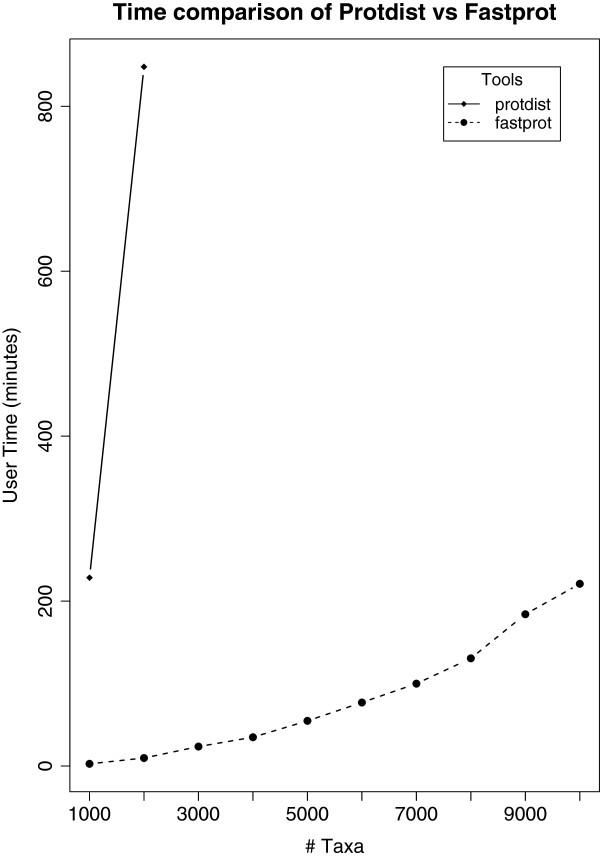
**Time comparison of ****fastprot****vs****protdist****.** This figure shows the time comparison between fastprot and protdist on 10 protein families with family size ranging from 1,000 to 10,000. The time duration for each experiment was limited to 24 hours. We can see that fastprot clearly outperforms protdist when computing distance matrices for protein families. protdist took 14.07 hours to compute the distance matrix for a protein family of size 2,000 while fastprot computed it in 0.16 hours.

fastprot estimates the evolutionary distance between aligned protein sequences. It implements two methods for calculating the distance between protein sequences: the maximum likelihood (ML), which for two aligned sequences *a* and *b* returns *argmax*_*d*_*Pr*(*a*,*b*∣*d*), and the expected distance, which returns *E*[*d*∣*a*,*b*] (see further [11]). The ML estimator uses Newton-Raphson method to find the optimum. It is, however, slower than the expectation estimator.

fastprot provides more distance functions when compared to other neighbour joining tools considered in this study. Clearcut (version 1.0.9) [9], Ninja (version 1.2.1) [8], and RapidNJ (version 2.1.0) provide JC [17] and JCK [18], QuickTree (version 1.1) [5] provides JCK, while fastprot implements seven distance functions including JC, JCK, WAG [20], JTT [21], DAY [22], MVR [23], and LG [24]. It is more than 200 times faster than protdist, a Phylip program for estimating distance matrices for protein alignments (see Figure 2). fastprot also allows longer accessions compared to protdist which has the limitation of ten characters for an accession number.

In addition to fastprot, we introduce fastprot_mpi, an implementation of fastprot using MPI libraries. fastprot_mpi can scale linearly to the number of nodes available on a cluster (see Table 1) and can handle very large protein families.

**Table 1:** **Time Comparison of ****fastprot****vs****fastprot_mpi**

**Tools**	**Nodes**	**Time (minutes)**
fastprot	1	1149
fastprot_mpi	8	148
fastprot_mpi	16	76
fastprot_mpi	32	40
fastprot_mpi	64	22

A protein family of 5186 family members was considered for this analysis, and time (rounded to the whole minutes) was recorded for each experiment using the Unix command "time". All experiments were performed on node level at a cluster machine. There were 8 cores in single node of the cluster machine on which the experiments were performed.

### Features of fnj

The fnj program implements three tree reconstruction methods, and the default is FNJ [14]. Furthermore, Neighbor-Joining [13], the mainstay of phylogenetics, as well as the more recent improvement BioNJ [1], are available as command line options. The program supports the formats used by fastdist and fastprot (i.e. XML and PHYLIP).

### Bootstrap feature

Bootstrap analysis is built into fastdist, fastprot, fastprot_mpi and does not require a separate program. Users can generate multiple bootstraps of the same dataset and store it to a file. By default, we keep a distance matrix from the original dataset along with the distance matrices from multiple bootstrap datasets. However, if users are interested in keeping the distance matrices only from bootstrap datasets, they can use the option -k, which will ignore the distance matrix from the original dataset and compute the distance matrices only for bootstrap datasets. In fact, users can run parallel jobs for each bootstrap in a distributed environment. For instance, if you want to infer phylogenetic trees for each of the 100 bootstraps of original dataset (say Input.fasta), you can use the GNU parallel[25] command in the following manner:

We provide a random seed option -s for the reproducibility of results. If a random seed option is not specified, the program will use the current time stamp for bootstrap analysis.

## Results and discussion

In order to access the performance of fastphylo compared to other NJ-based tools, we considered two performance metrics: speed and memory utilization. Apart from this, we were also interested in measuring how large gene families fastphylo can handle. The basic motivation for such analysis comes from the limitation that most of the NJ tools fail to compute phylogenetic trees for very large gene families.

### Simulated data

To evaluate the performance of fastphylo, we simulated two different datasets. The first dataset, which we called dataset-1, consists of 10 gene families with family size ranging from 1,000 to 10,000 family members. The second dataset, dataset-2, contains 20 gene families with gene sequences ranging from 5,000 to 100,000. Each gene sequence is 2,000 nucleotides long, while each protein sequence is 350 amino acids long.

We used tools developed by our colleagues Ali Tofigh and Bengt Sennblad to generate trees and sequences. All the details on parameter settings for generating trees and sequences are mentioned in Additional file 1.

### Environment and experimental set-up

We used Clearcut, QuickTree, Ninja, and RapidNJ as references in our experiments. QuickTree and Clearcut are implemented in C, RapidNJ and fastphylo are implemented in C++ while Ninja is implemented in Java. Ninja, QuickTree, and RapidNJ reduce running time of the canonical NJ method while fnj and Clearcut modify the original NJ criteria in order to gain computational speed. Since we took aligned DNA sequences as an input and Newick formatted trees as an output, we used the command

for all our experiments. All experiments were performed on a cluster machine. Each cluster node has 8 cores and each core has 3 GB of RAM. We set up two experimental environments: one for dataset-1 and one for dataset-2, separately. For dataset-1, we ran each experiment on a single dedicated core with a time duration of 2 hours for each job. However, for dataset-2, the time limit for each experiment was set to 24 hours, and each experiment was performed on a node instead of a core due to memory requirements.

We used Massif, a memory profiling tool available in the Valgrind suite [26], to profile memory consumption of the aforementioned NJ tools. The standard time tool available in Linux (version 2.6.32) was used for measuring running time of each experiment. Only "User time" output from the time tool is considered in the time comparison analysis. We tried to use the best performance parameters for each tool in our analysis. All the details on the choice of parameters used, for different NJ tools, are mentioned in Additional file 1.

### Results on dataset-1

**Figure 3:**
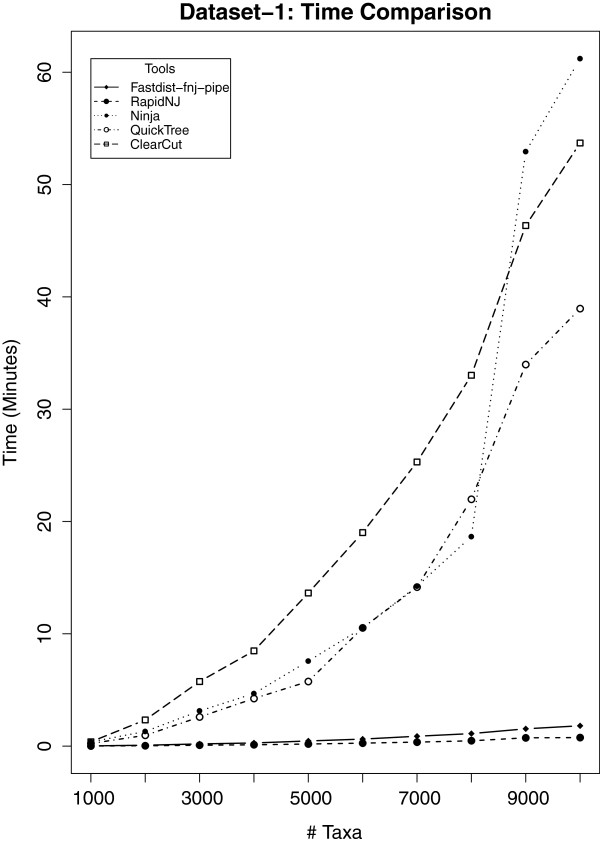
**Time comparison analysis for ****dataset-1****.** Ten gene families were considered for this analysis, with family size ranging from 1,000 to 10,000. Vertical axis represents 'User time’ while horizontal axis represents the number of gene sequences per gene family. RapidNJ is slightly faster then fastdist-fnj pipe.

**Figure 4:**
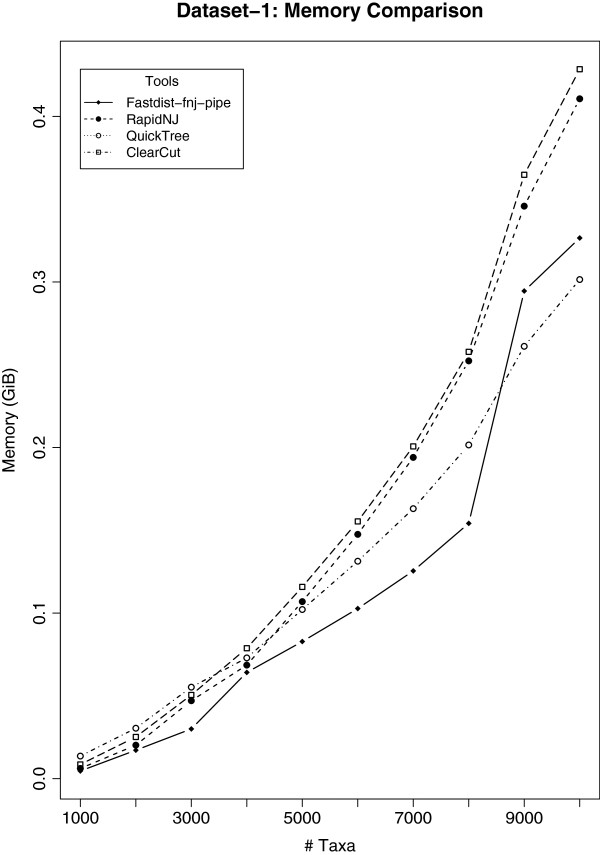
**Memory comparison analysis for ****dataset-1****.** Ten gene families were considered for this analysis, with family size ranging from 1,000 to 10,000. The vertical axis represents memory utilization in GiB’s, while the horizontal axis represents the number of gene sequences per gene family. The results here suggests that ClearCut and RapidNJ use more memory compared to the rest of NJ tools as the family size increases.

Results on dataset-1 is shown in Figures 3 and 4. Figure 3 shows the time comparison analysis, while in Figure 4 we show the memory utilization of different NJ tools considered in our study. From the time comparison in Figure 3, it is clear that our fastdist-fnj pipe outperforms Clearcut, QuickTree and Ninja, while it shows a slight delay when compared with RapidNJ. To investigate this further, we ran the same experiment on dataset-2 (see section 'Results on dataset-2’).

### Results on dataset-2

**Figure 5:**
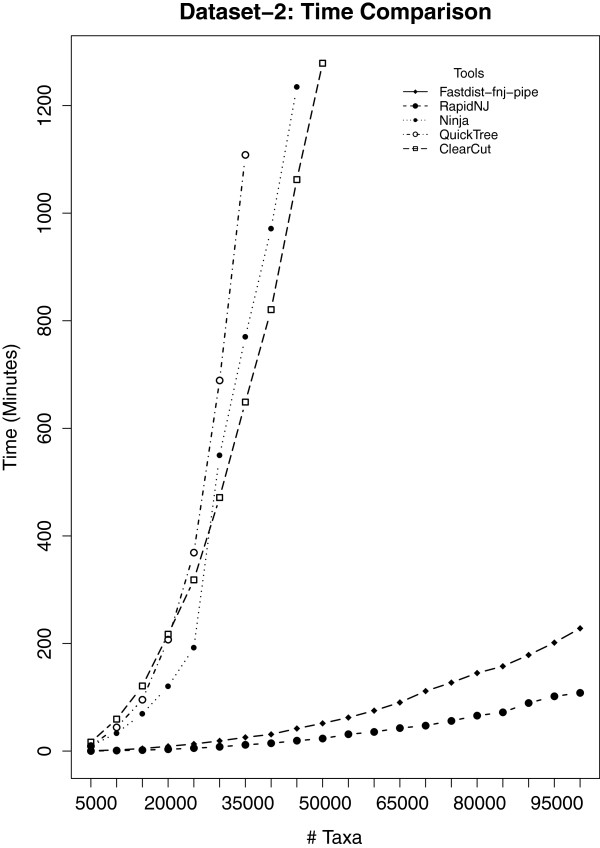
**Time comparison analysis for ****dataset-2****.** Twenty gene families were considered for this analysis, with gene family members ranging from 5,000 to 100,000. The results suggest that fastphylo (fastdist-fnj-pipe) outperforms all the NJ-based tools considered in this study, except RapidNJ. QuickTree took the longest time to compute phylogenetic trees. In the allowed experimental time, i.e. 24 hours, QuickTree was able to compute phylogenetic trees for 7 gene families of size ranging from 5,000 to 35,500 family members, Ninja did it for 9 gene families with family members limited to 45,500, and ClearCut computed trees for 10 gene families (family size limited to 50,000). RapidNJ and fastphylo (fastdist-fnj-pipe), however, computed phylogenetic trees for all the 20 gene families considered in this study, in a reasonable time.

**Figure 6:**
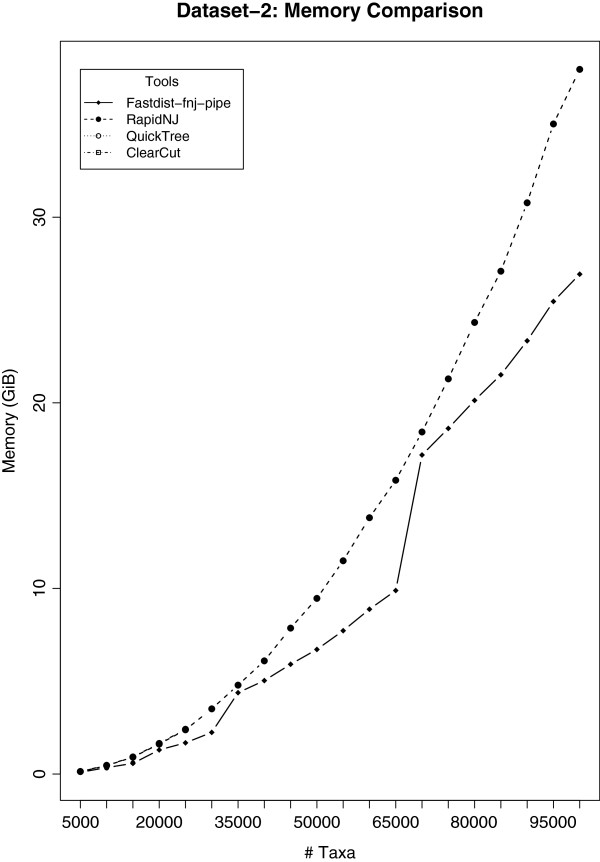
**Memory comparison analysis for ****dataset-2****.** Twenty gene families were considered for this analysis, with gene family members ranging from 5,000 to 100,000. Results are similar to Figure 5. For large phylogenies, RapidNJ uses much more memory as compared to fastphylo (fastdist-fnj-pipe). However, we cannot explain the source of sudden jumps in memory utilization of fastphylo.

In order to address the question of how large gene families fastphylo can handle and also to investigate the delay in Figure 3, we ran the fastdist-fnj pipe and other NJ tools on dataset-2. The results are formulated in Figures 5 and 6. Both fastdist-fnj pipe and RapidNJ computed phylogenetic trees for all the 20 gene families of size ranging from 5,000 to 100,000, while ClearCut, Ninja and QuickTree only computed phylogenetic trees for gene families of size ranging from 5,000 up to 50,000, 45,500 and 35,500, respectively, in the allocated time, i.e. 24 hours. The graph in Figure 5 reveals that RapidNJ is ∼2 times faster than fastdist-fnj pipe. However, Figure 6 shows that the fastdist-fnj pipe uses less memory (a factor of ∼1.5) than RapidNJ.

**Figure 7:**
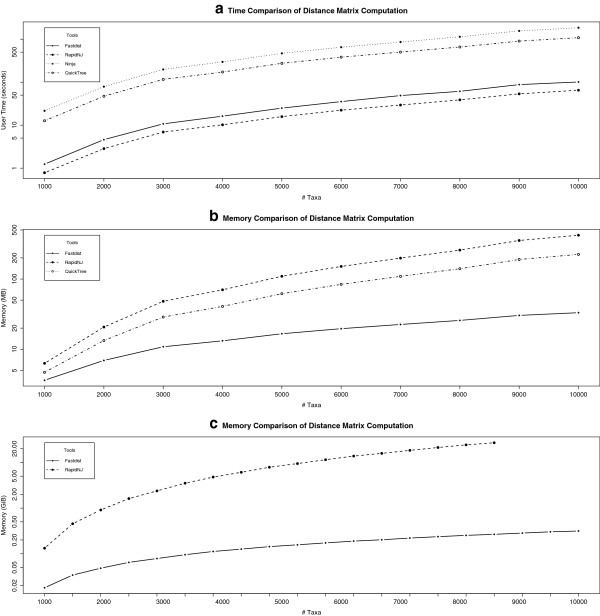
**Time and memory comparison of the distance matrix computation.** The analysis in Figure 7**a** and 7**b** were performed on dataset-1, while Figure 7**c** shows the memory utilization of RapidNJ and fastdist (using the binary format) on dataset-2. ClearCut was not considered in this experiment since it outputs the distance matrix as an option, and at the same time it outputs the phylogenetic tree.

**Figure 8:**
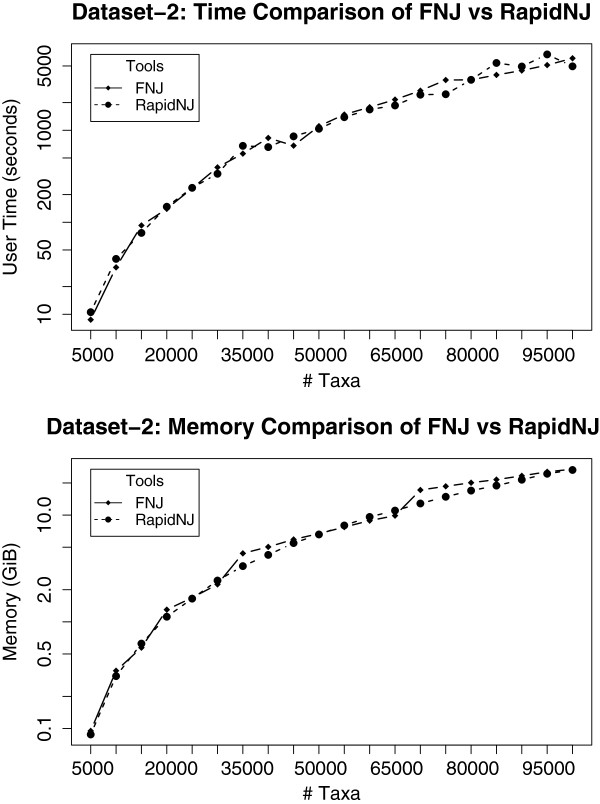
**Time and memory comparison between ****fnj**** and ****RapidNJ****.** This analysis was performed on dataset-2. fnj and RapidNJ both performed almost similar on the time analysis. Memory consumption figure shows that fnj uses slightly more memory in certain cases but the overall difference is not large.

To further investigate the delay in fastdist-fnj pipe, we split the experiment into two phases: 1) compute the distance matrix separately; and 2) compute the phylogenetic tree using the distance matrix as an input to the neighbour joining tools considered in this study. The results of these investigations are formulated in Figures 7 and 8, respectively. Figure 7 shows the time and memory comparison of NJ tools for computing the distance matrices. It is evident that RapidNJ outperforms all the other tools. It is ∼2 times faster than fastdist (see Figure 7a). However, RapidNJ’s memory consumption increases quadratically with the number of sequences, while fastdist’s memory utilization increases linearly with the number of sequences (see Figure 7c). In Figure 7c, we report the results of RapidNJ upto 85,000 taxa. This is due to the memory limitation for computing the distance matrices for this experiment, i.e. 24 GB RAM. RapidNJ computed distance matrices for 17 gene families of size ranging from 5,000 to 85,000 sequences, while fastdist computed distance matrices for all the 20 gene families of size ranging from 5,000 to 100,000 sequences within the allocated memory. We can attribute the delay in the fastdist-fnj pipe, when compared to RapidNJ, in Figures 3 and 5 to the slow computation of distance matrices by fastdist program.

Figure 8 shows the time and memory comparison of fnj and RapidNJ. The input to both programs is distance matrices. We used output from fastdist in a binary format as an input to fnj, and distance matrices in PHYLIP format to RapidNJ. It is interesting to note that fnj and RapidNJ performed similar on both the time and memory comparison analysis, but RapidNJ has an advantage on memory usage.

## Conclusions

FastPhylo is a software package containing software that is easy to use and has well-defined interfaces. It is an efficient software that enables very large problem sizes. In addition, Fastphylo can be a good tool of choice in many studies: for instance, in MCMC and maximum likelihood (ML) methods for phylogeny reconstruction, it can be used to generate a good starting tree. Further more, Fastphylo’s modular architecture offers maximum flexibility in phylogenetic computations.

## Availability and requirements

**Project name:** Fastphylo

**Project home page:**http://fastphylo.sourceforge.net

**Operating system(s):** Linux, Mac OS X (10.6.8 and 10.8.4)

**Programming language:** C++

**Licence:** MIT License

**Any restrictions to use by non-academics:** None

## Competing interests

The authors declare that they have no competing interests.

## Authors’ contributions

MAK, IE, ES, KN, RVG, RS and PS developed the software; MAK and LA designed and performed the analysis; MAK and LA wrote the manuscript. All the authors read and approved the final manuscript.

## Supplementary Material

Additional file 1 Supplementary material for fastphylo: fast tools for phylogenetics. Click here for file
