# Supplementary material for: Fastphylo: Fast tools for phylogenetics
Source: BMC Bioinformatics. 2013 Nov 20;14:334. doi: 10.1186/1471-2105-14-334 (PMC4225504; doi:10.1186/1471-2105-14-334)
Supplement: Additional file 1 — Supplementary material for fastphylo: fast tools for phylogenetics. [file 1471-2105-14-334-S1.pdf]

Supplementary Material for fastphylo: Fast tools for phylogenetics

Mehmood Alam Khan , Isaac Elias, Erik Sjölund, Kristina Nylander, Roman Valls Guimera, Richard Schobesberger, Peter Schmitzberger, Jens Lagergren and Lars Arvestad

Contents

1 Synthetic Data Generation **2**

1.1 Trees Generation 2

1.2 Sequence Generation 2

2 Parameter Settings for NJ Tools **3**

3 Required Packages for `Fastphylo` **3**

1 Synthetic Data Generation

1.1 Trees Generation

We used the `phyltr-gen-stree` software tool for generating synthetic phylogenies. `phyltr-gen-stree` is an in-house software tool, developed by our colleague Ali Tofigh, to generate species trees using a stochastic birth-death process. We used the following command to generate trees for `dataset-2`:

```
phyltr-gen-stree --min-size <integer> --max-size <integer> 500 14.5
3 -s -a 100000
```

Where,
`--min-size` is the minimum number of leaves in a species tree.
`--max-size`: the maximum number of leaves in a species tree.
`-s`: birth-death process starts at speciation.
`-a`: the maximum number of times the process will run if the species goes extinct.
Time.
Birth rate: 14.5
Death rate: 3

We used a similar shell script for `dataset-1` but with varying `--min-size` and `--max-size`.

1.2 Sequence Generation

To generate DNA and protein sequences, we used `beep_generateSeqData` developed by Bengt Sennblad. `beep_generateSeqData` is a part of the PrIME software suite available at http://prime.sbc.su.se. The choice of parameters used for generating sequences are as follows:

```
beep_generateSeqData -Sm JTT -Sa -Gu -Em iid -Ed Gamma -El <treefile>
<nchars>
```

Where,
`-Sm` is the substitutional model. We used `JTT` substitutional model for generating protein sequences while for DNA sequences, we considered `JC69`.
`-Sa` refers to alpha, the shape parameter of the Gamma distribution modelling site rates. We used the default alpha i.e. 1.
`-Em` is the edge rate model.
`-Ed` is the density function to use for edge rates.
`-El` refers to generate edge-lengths directly from rate model.
`treefile` refers to the path containing input tree in newick format.
`nchars` refers to the sequence length. For DNA sequences, we set it to 2000 while for protein sequences, we used 350.

2 Parameter Settings for NJ Tools

We used the Kimura substitution model for computing the distance matrices in all our experiments. Below here are the commands we used for all the NJ tools considered in this study:

Fastphylo:
```
fastdist -I fasta input.fasta -O binary | fnj -I binary -O newick -o output.newick
```

RapidNJ:
```
rapidnj -i sth -o t -k 10 -t d input.stockholm > output.newick
```

QuickTree:
```
quicktree -kimura -in a input.stockholm > output.newick
```

ClearCut:
```
clearcut --kimura --alignment -D --in=input.fasta --out=output.newick
```

Ninja:
```
ninja --alph_type=d --in_type a input.fasta --out_type t -o output.newick
```

3 Required Packages for `Fastphylo`

To install `Fastphylo` on Ubundu or Mac OSX, you need to pre-install the following packages:

1. cmake
2. libxml2
3. BLAS
4. LAPACK
5. openmpi
6. wget
